# From clinical fluids to environmental matrices: quantitative monitoring of infectious monkeypox virus based on PMA-qPCR during the Guangdong outbreak

**DOI:** 10.1186/s41182-026-01028-z

**Published:** 2026-07-15

**Authors:** Jianhai Yu, Xin Zhang, Xiaoting Xie, Yutian Miao, Shen Huang, Li Zhu, Yan Zhan, Yifan Lin, Changyun Sun, Linqing Wang, Penghui Jia, Qinghua Wu, Weiwei Xiao, Bao Zhang, Chenguang Shen, Baisheng Li, Wei Zhao

**Affiliations:** 1 BSL-3 Laboratory (Guangdong), Guangdong Provincial Key Laboratory of Tropical Disease Research, Key Laboratory of Infectious Diseases Research in South China, School of Public Health, Southern Medical University, No. 1023, South Shatai Road, Baiyun District, Guangzhou, 510515 Guangdong Province China; 2 Institute of Microbiology, Center for Disease Control and Prevention of Guangdong Province, No. 160 Qunxian Road, Dashi Street, Panyu District, Guangzhou, 511430 Guangdong Province China; 3 Guangdong Provincial Key Laboratory of Pathogen Detection for Emerging Infectious Disease Response, No. 160 Qunxian Road, Dashi Street, Panyu District, Guangzhou, 511430 Guangdong Province China; 4 Guangzhou Center for Disease Control and Prevention, No.1 Qide Road, Baiyun District, Guangzhou, 510440 Guangdong Province China

**Keywords:** Infectious monkeypox virus, PMA-qPCR, Outbreak surveillance, Quantitative monitoring, Clinical and environmental matrices, Viral decay kinetics

## Abstract

**Background:**

Mpox, caused by monkeypox virus (MPXV), remains a public health priority. Standard qPCR detects viral DNA but cannot distinguish infectious virions from residual genetic material of inactivated virus, complicating transmission risk assessment. This study aimed to develop a rapid assay for quantifying infectious MPXV across clinical and environmental matrices in Guangdong Province, China.

**Methods:**

We developed a propidium monoazide-assisted qPCR (PMA-qPCR) assay that selectively amplifies DNA from intact virions, and embedded it within the Guangdong provincial Center for Disease Control and Prevention surveillance network. We examined 23 clinical fluid specimens from 15 mpox patients, 337 surface swabs and 53 domestic wastewater samples from patient living environments, and 498 terminal wastewater samples from flights and sewage treatment plants collected between June 2023 and August 2024. Decay kinetics were evaluated at 25 °C and 30 °C under varying pH, with initial viral loads of 10^3^ and 10^4^ PFU/mL.

**Results:**

PMA-qPCR discriminated infectious from non-infectious virus. Co-incubation with 50 μM PMA and 0.005% SDS effectively blocked amplification of non-infectious DNA, and the assay showed quantitative concordance with the standard plaque assay. Infectious MPXV loads were highest in skin lesions and anal swabs, exceeding throat swabs by more than 100-fold. Among surface samples, 41.54% (140/337) tested positive for MPXV DNA by qPCR, but only 25.71% (36/140) of these contained infectious virus at low loads (126.79 ± 93.57 PFU/mL). For wastewater, 11.32% (6/53) of domestic samples from patient living environments tested positive by qPCR, yet no infectious MPXV was detected. No MPXV DNA was detected in 498 terminal wastewater samples from flights or sewage treatment plants. Decay experiments demonstrated that when the initial concentration was 10^3^ to 10^4^ PFU/mL, infectious MPXV decreased by more than 99% within 48 h, with accelerated decay under acidic pH and elevated temperature, particularly at lower initial concentrations.

**Conclusions:**

PMA-qPCR provides a practical tool for rapid cross-matrix quantification of infectious MPXV. Direct contact with skin lesions or anal secretions likely dominates transmission, whereas environmental persistence is limited. Testing for infectious virus within 48 h of collection is recommended, as infectious MPXV decays by over 99% within this window.

**Supplementary Information:**

The online version contains supplementary material available at 10.1186/s41182-026-01028-z.

## Introduction

Mpox, caused by monkeypox virus (MPXV), was declared a Public Health Emergency of International Concern (PHEIC) by World Health Organization (WHO) in 2022 and again in 2024 following the emergence of clade I in Africa [1–3]. As of November 30, 2025, a cumulative count of 175,415 mpox cases and 467 fatalities had been reported from 142 countries and regions worldwide. Faced with this ongoing challenge that poses a public health risk internationally, there is an urgent need for a coordinated response to curb the wider spread of mpox [4].

During the ongoing epidemic, MPXV predominantly spreads through direct or sexual contact, with skin lesions and anogenital ulcers carrying the highest viral loads [5–7]. However, although MPXV DNA is frequently detected in various body fluids, including 60–70% of anal and throat swabs, 50% of semen, and 20% of blood and urine samples, a positive PCR does not necessarily indicate infectiousness, as some skin lesions yield DNA without viable virus [8–10]. Recent studies have demonstrated that MPXV stability varies markedly across media. The virus persists for weeks in proteinaceous fluids such as blood and semen, whereas it decays more rapidly in saliva, urine, and feces [11]. This medium-dependent persistence complicates the interpretation of PCR-positive results, as viral DNA may remain detectable long after infectious virions have been inactivated. Consequently, the distribution of infectious MPXV across different body fluids and its true environmental persistence remain poorly characterized.

Beyond clinical specimen testing, understanding viral infectivity in non-clinical specimens is essential for comprehensive infectivity profiling. Environmental surveillance of patient-contact surfaces and wastewater has emerged as a complementary tool for tracking viral circulation [12–14]. Currently, MPXV DNA has been detected on household surfaces [15–17] and in wastewater [18–21] from multiple outbreak-affected countries. The latest research indicates that wastewater monitoring can serve as a beneficial supplement to case monitoring to reflect the current situation of mpox transmission [22, 23]. Notably, Yinda et al. demonstrated that infectious MPXV can remain viable in untreated wastewater for multiple days and proposed that testing for infectious MPXV could be a valuable complement to PCR-based wastewater surveillance when high levels of viral DNA are detected [11]. However, these studies primarily recorded DNA presence without systematically quantifying infectious virus across the full transmission chain.

Since September 2022, mpox has disseminated across more than 30 provinces in China, with 3,436 cumulative cases reported as of August 2025. Guangdong Province was among the first areas to report cases and initiate wastewater surveillance [24]. Yet the dynamic characteristics of infectious MPXV throughout the environmental transmission pathway, from clinical fluids to wastewater, remain notably limited. Moreover, because MPXV persistence varies substantially across media [11], monitoring strategies may need to differ by specimen type. Clinical fluids require assessment of ongoing shedding, whereas environmental samples must distinguish residual nucleic acid from viable virus. Existing nucleic acid amplification and serology techniques cannot determine MPXV infectivity [25–27], and cell culture plaque assays are too slow and resource-intensive for routine outbreak response [28]. Therefore, a rapid molecular method capable of quantifying infectious MPXV across diverse clinical and environmental matrices is urgently needed.

**Fig. 1:**
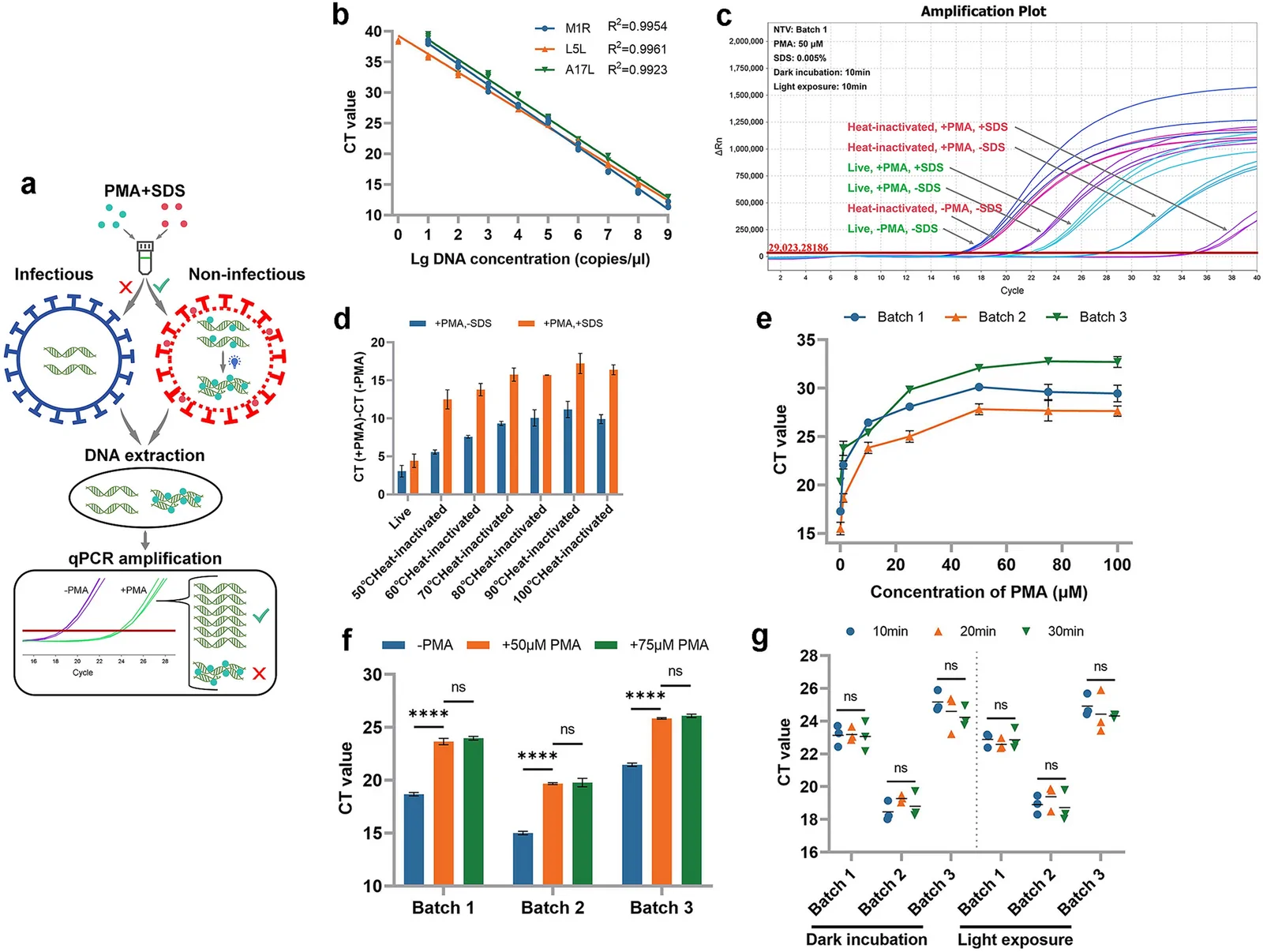
The construction of a PMA-assisted qPCR assay for rapid quantitative detection of infectious monkeypox virus. **a** The mechanism of PMA-assisted qPCR assay for distinguishing infectious and non-infectious MPXV. **b** Standard curves for qPCR detection targeting the M1R, L5L, and A17L genes of MPXV, respectively. **c** The effect of PMA and SDS on blocking PCR amplification of non-infectious NTV DNA. **d** The combined effect of 50 μM PMA and 0.005% SDS on qPCR detection of heat-inactivated dead NTV. **e** Exploration of the optimal working concentration of PMA on qPCR detection of heat-inactivated dead NTV. **f** The effect of PMA with 50 μM and 75 μM concentration on qPCR detection of infectious NTV in live virus cell culture. **g** Exploring the effect of different dark incubation and blue light exposure times on qPCR detection of infectious NTV in live virus cell culture

Here, we developed a propidium monoazide (PMA)-assisted quantitative polymerase chain reaction (qPCR) assay (abbreviated as PMA-qPCR) that is widely used to detect viable bacteria and has been verified in the environmental detection of infectious bacteria and viruses [29, 30]. This assay employs membrane-destabilizing agent sodium dodecyl sulfate (SDS) to disrupt the dead viral envelope, enabling PMA to penetrate MPXV and react covalently with MPXV DNA under blue light. The resulting PMA–DNA conjugate can interrupt PCR amplification and effectively minimize non-infectious viral DNA contamination, thereby ascertaining rapid quantitative detection of infectious MPXV (Fig. 1a). To validate this tool for integrated clinical and environmental surveillance, we embedded PMA-qPCR within the routine outbreak response system of Guangdong Province, China. We examined 23 clinical fluid specimens from 15 mpox patients, 337 object surface swabs and 53 domestic wastewater samples from patient living environments, and 498 terminal wastewater samples from inbound flights and sewage treatment plants collected through the Center for Disease Control and Prevention (CDC) surveillance network from June 2023 to August 2024. This design allowed us to simultaneously (i) validate the assay across the full transmission spectrum from human shedding to fomite contamination and terminal wastewater, and (ii) report real-world viral distribution and infectivity patterns during an active outbreak in a major Chinese province, thereby providing actionable data for both patient management and environmental monitoring strategies.

## Methods

### Cells and viruses

BHK-21 and Vero-E6 cells used for virus culture were preserved in our laboratory. The non-replicating Tiantan vaccine virus (NTV) was cultured using BHK-21 cells in our laboratory. The clinical isolates of MPXV from Guangdong were cultured using Vero-E6 cells in the Biosafety Level-3 (BSL-3) laboratory of Guangdong CDC, and the viral load of NTV and MPXV were detected by plaque assay and summarized in Table S2.

### Samples collection

The 23 different clinical fluid samples from 15 mpox patients (Historical publications cited in this study use the term 'monkeypox' as it appeared in the original literature, while the current manuscript adopts 'mpox' in accordance with the WHO-recommended nomenclature), including 9 throat swabs, 5 anal swabs, and 9 herpes collection fluid from skin lesions, were collected by the Guangdong CDC. The background information of all clinical samples is summarized in Table S2.

The environmental samples were collected by the Guangzhou CDC through routine surveillance protocols from June 19, 2023 to August 28, 2024, spanning a 14-month outbreak monitoring period, with aviation wastewater and flight surface samples randomly collected from inbound flights at Guangzhou Baiyun International Airport (5 flights randomly selected per week), sewage treatment plant wastewater systematically collected from 4 randomly selected plants per week in areas with reported mpox cases to ensure continuous community-level surveillance, and patients’ living environment samples (objects surface and domestic wastewater samples) collected from 59 mpox patients who were randomly selected from confirmed cases throughout the surveillance period rather than from a single outbreak cluster. For wastewater samples, the virus was enriched and concentrated using the polyethylene glycol precipitation method in WS T 799-2022, and detected for MPXV using qPCR. Samples of 11 object surfaces were collected according to the standard DB32/T 3762.8-2020 and their sampling cotton swabs were ultimately stored in a virus sampling tube containing 3 mL of virus preservation solution to minimize surface-to-surface variability that might otherwise affect PMA penetration or DNA extraction efficiency.

### Identification of target genes for monkeypox virus qPCR detection

Utilizing the GenBank database, the functional annotation of the coding protein gene of MPXV was summarized through MPXV (GenBank: NC_063383.1), vaccinia virus (GenBank: M30502.1 and NC_006998.1) and cowpox virus (GenBank: NC_003663.2), and the gene location was carried out in Guangdong MPXV (CNCB: C_AA038892.1) previously reported by our laboratory [24] to identify the surface membrane proteins related to MPXV infection. Further, we used the previously reported global MPXV genetic diversity sequence dataset [24] in our laboratory to conduct conservative analysis on the surface membrane proteins identified by functional annotation, and obtained the membrane proteins that were completely unmutated during the global transmission process of MPXV clade IIb and its derived lineages.

### PMA-qPCR assay

The pretreatment procedure for PMA is as follows: add 50 μM PMA and 0.005% SDS to the sample, incubate in the dark for 10 min and expose to blue light under a blue light excitation instrument for 10 min. These concentrations and processing times were optimized using live and heat-inactivated NTV cell culture fluids to ensure that 0.005% SDS facilitates 50 µM PMA penetration of compromised envelopes without affecting intact infectious virions, and subsequently validated for clinical MPXV isolates as described below. Then, virus DNA was extracted from samples treated with or without PMA using the NucleoSpin Virus kit (Macherey–Nagel, Germany, Code No. 740983), and was quantified by qPCR detection using the Pro Taq HS Premix Probe qPCR Kit (Accurate Biotechnology (Hunan) Co., Ltd, China, Code No. AG11704) with the amplification protocol of 95 ℃ for 30s, 95 ℃ for 5s, 60 ℃ for 30s, for 40 cycles. qPCR detection and data collection were performed using QuantStudio 6 instrument (Thermo, America). Primer and probe sequences are provided in Table S1.

### Decay kinetics of monkeypox virus in wastewater

Domestic wastewater samples from patients' living environments (*n* = 50), flights (*n* = 50), and sewage treatment plants (*n* = 50) were randomly selected, and their pH values were measured. Three flight wastewater samples (pH = 8–8.5) and three sewage treatment plant wastewater samples (pH = 6–6.5) were centrifuged to remove suspended solids and precipitates, thereby eliminating potential interference for PMA-qPCR process from virus adsorption to particulate matter. MPXV was then spiked into wastewater at final concentrations of 10^3^ and 10^4^ PFU/mL and incubated at 25 °C and 30 °C, respectively, with an MPXV control (virus solution without wastewater) and a negative control (wastewater containing only culture medium) included in parallel. Samples were collected at 24 and 48 h for measurement of MPXV DNA and infectious MPXV loads.

### Statistical analysis

Data visualization was performed using GraphPad Prism v9.5.1, and all statistical analyses were conducted using SPSS v25.0. Independent sample t-tests were used to evaluate the effects of PMA concentration, dark incubation time, and blue light exposure time on PMA-qPCR performance. Paired sample t-tests were used to compare measurements between PMA-qPCR and standard qPCR. For decay kinetics experiments, data are presented as mean ± standard deviation (SD). Independent sample t-tests were also used to compare pH values across wastewater sources. Prior to repeated-measures ANOVA of decay kinetics, Shapiro–Wilk normality tests and Levene/Brown–Forsythe homoscedasticity tests were performed (all *P* > 0.05). Geisser–Greenhouse correction was applied when the sphericity assumption was violated. Paired sample t-tests were used to assess the effects of sample collection source, pH, and incubation temperature on MPXV decay. Statistical significance was defined as follows: ns *P* > 0.05; *P < 0.05; **P < 0.01; ***P < 0.001; and ****P < 0.0001.

## Results

### Development of the PMA-qPCR assay

Infectious MPXV is an enveloped virus that enters cells via membrane or surface proteins. Gene annotation identified 40 membrane or surface proteins among 192 MPXV protein-coding genes (Figure S1 and Table S3). Nineteen of these remained completely unmutated throughout global clade IIb transmission, making them suitable targets for infectious MPXV detection (Table S3 and Table S4). Combining gene length and nucleotide composition analyses, we tailored qPCR primers and probes for OPG95, OPG104, and OPG143, encoding M1R, L5L, and A17L, respectively (Table S1). Notably, the three selected genes are also highly conserved in clade Ib evolution, with primer and probe binding regions remaining mutation-free across clade I and II evolution. Standard curves showed that L5L targeting provided greater stability (*R*^2^ = 0.9961) and sensitivity (Fig. 1b), making it our detection target.

To assess PMA activity safely, the engineered primers and probes recognize the conserved L5L gene in NTV. For NTV from cell culture, qPCR cycle threshold (CT) values of both active and heat-inactivated fluids increased substantially after PMA (50 μM) pretreatment, with heat-inactivated virus showing a higher ΔCT (CT(+ PMA) − CT(− PMA)) than active virus. However, heat-inactivated virus still showed relatively lower CT values, indicating that PMA reduced non-infectious DNA interference but failed to fully permeate impaired particles (Fig. 1c). Therefore, SDS was incorporated with PMA, revealing that 0.005% SDS enabled efficient PMA penetration and blocked PCR amplification of non-infectious NTV DNA in heat-inactivated samples (Fig. 1c and d). Exploration of optimal PMA conditions showed that CT values for three batches of heat-inactivated NTV gradually increased with PMA concentration, peaking at 50 μM before plateauing (Fig. 1e and f). Comparable CT values for live NTV at 50 µM and 75 µM PMA indicated that the selected concentration does not damage intact viral envelopes and therefore avoids false negatives for infectious virus detection (Fig. 1f). Subsequent dark incubation and blue-light exposure times did not affect PMA action within the 10–30 min range (Fig. 1g). These findings indicated that co-incubation with 50 μM PMA and 0.005% SDS for 10 min in the dark, followed by 10 min blue-light exposure, effectively discriminates infectious from non-infectious NTV.

### Verification of the accuracy of the PMA-qPCR assay

**Fig. 2:**
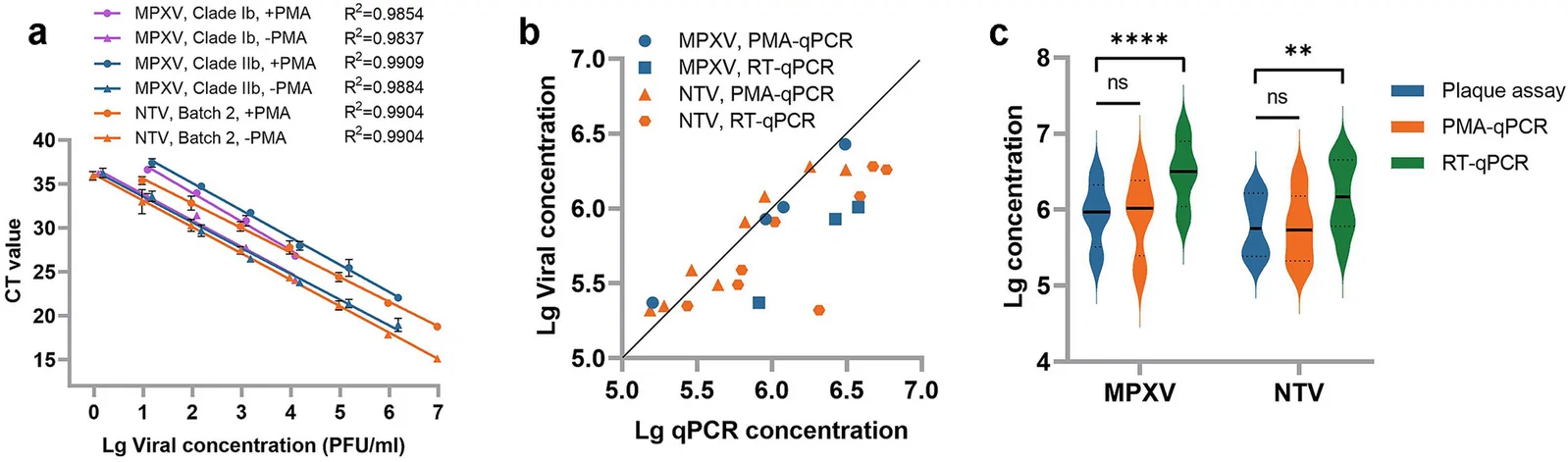
The verification of the accuracy of PMA-qPCR assay and plaque assay for detecting infectious MPXV and NTV. **a** Standard curves for detecting infectious MPXV and NTV in live virus cell culture using PMA-qPCR assay with 50 μM PMA and 0.005% SDS incubated in dark for 10 min and exposed to blue light for 10 min. **b** Consistency analysis between infectious MPXV and NTV measured by PMA-qPCR and live viral load measured by gold standard plaque assay in live virus cell culture. The black diagonal represents the equation Y = X, and the closer the scattering points are to the diagonal, the stronger the consistency between PMA-qPCR results and plaque assay. **c** The differences between the results of PMA-qPCR or qPCR and plaque assay in live virus cell culture

The standard curves of active MPXV and NTV from cell culture revealed a good linear relationship between active virus concentration and CT values, with or without PMA. The standard curve with PMA exhibited a striking upward shift compared to that without PMA, while maintaining a notable parallel relationship. Intriguingly, PMA treatment increased the limit of detection (LOD) of qPCR, such that in the absence of PMA, the LOD of viral fluids was 1–10 PFU/mL, but 10–100 PFU/mL post-PMA pretreatment (Fig. 2a). Nevertheless, when multiple batches of MPXV and NTV were tested, virus concentrations measured without PMA were considerably higher than plaque assay titers due to non-infectious DNA interference. In contrast, PMA-qPCR infectious virus concentrations showed adequate concordance with plaque assay results (Fig. 2b and c). Notably, the concordance between PMA-qPCR and plaque assay results for clinical MPXV isolates supports the applicability of the NTV-optimized conditions to wild-type MPXV, although full parameter optimization was not performed directly on MPXV.

### Distribution characteristics of MPXV DNA and infectious MPXV in clinical body fluids

**Fig. 3:**
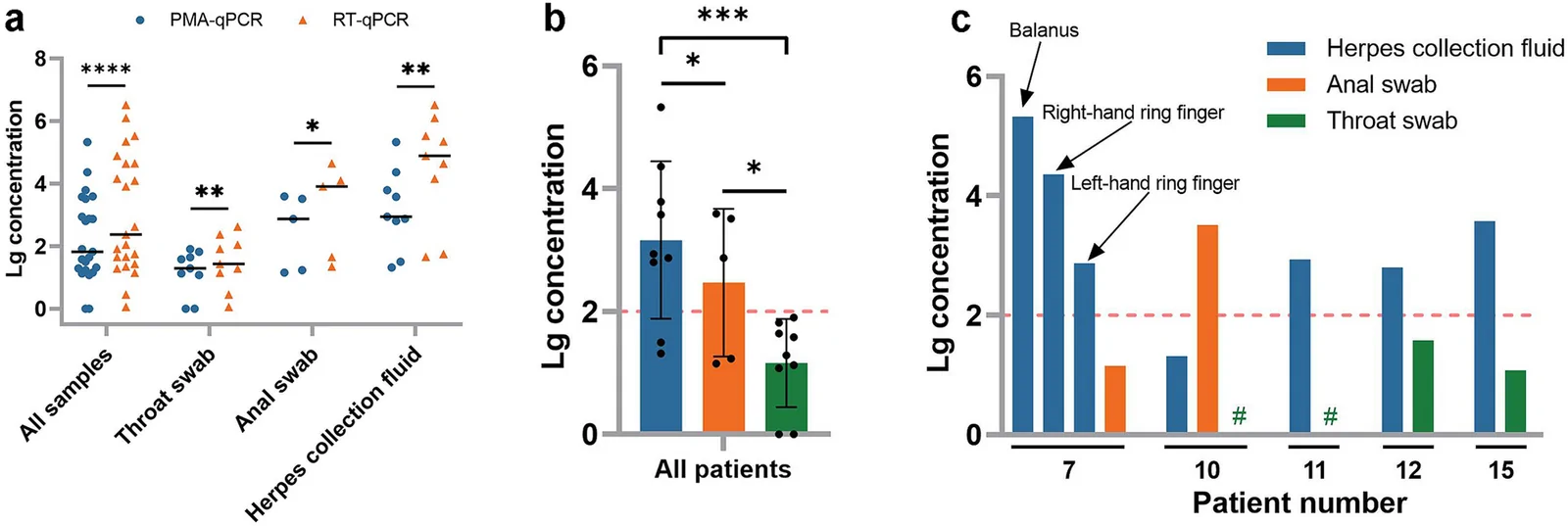
The application of PMA-qPCR assay for monitoring of infectious monkeypox virus in clinical body fluids. **a** The differences between infectious virus DNA measured by PMA-qPCR and the total virus DNA measured by qPCR in clinical body fluid samples of mpox patients. **b** Differences in the concentration of infectious MPXV in different types of body fluid samples from mpox patients. **c** Distribution characteristics of infectious MPXV in multiple body fluid samples collected from the same mpox patient

PMA-qPCR detection showed that the measured levels of infectious MPXV DNA in throat swabs, anal swabs, and herpes collection fluid from skin lesions were all lower compared to qPCR without PMA, with infectious DNA accounting for less than 1% of total DNA in some herpes collection fluid samples (Fig. 3a). Among all samples, the infectious MPXV titer was highest in herpes collection fluid, followed by anal swabs, while the lowest was throat swabs (Fig. 3b). There were variations in the concentration of infectious MPXV across multiple body fluids collected from the same patient. For example, in Patient 7, herpes collection fluid from glans lesions had a higher concentration compared to that acquired from the finger; in Patient 10, anal swab exhibited a higher concentration compared to herpes collection fluid. Throat swabs had the lowest concentration among the various body fluids of all patients; infectious MPXV could not be detected in two patients, while DNA could be detected by qPCR without PMA (Fig. 3c).

### Monitoring of MPXV in environmental samples related to patients' daily activities

From June 19, 2023 to August 28, 2024, we collected 249 wastewater and 249 passenger channel surface samples from 39 inbound civil aviation flights, covering 4 continents, 23 countries, and 49 air routes (Table S5), as well as 220 samples of domestic wastewater from sewage treatment plants in areas with reported mpox cases, and 337 object surface swab samples and 53 domestic wastewater samples from the living environments of 59 mpox patients (Table S6). The results showed that PCR-positive samples were detected in the living environment in direct contact with patients, but could not be detected in the monitoring of flights and sewage treatment plants that could reflect the terminal of daily activities. For patients, 71.19% (42/59) had positive object surfaces, and 13.64% (6/44) had positive domestic wastewater. Among all living environmental samples, 41.54% (140/337) of surface samples and 11.32% (6/53) of domestic wastewater were able to detect MPXV DNA. Notably, MPXV DNA could also be detected on the surface of objects in patients with positive wastewater testing, but the wastewater testing of patients with positive surface of objects may not necessarily detect MPXV DNA (Table 1 and Table S6).

**Table 1:** Monitoring of MPXV DNA in environmental samples and quantitative determination of infectious MPXV in PCR-positive samples

	Surface of objects		Wastewater		
	Patient environment	Flight	Patient environment	Flight	Sewage treatment plants
Total	337	249	53	249	220
qPCR	41.54% (140/337)	0% (0/249)	11.32% (6/53)	0% (0/249)	0% (0/220)
PMA-qPCR	25.71% (36/140)	–	0% (0/6)^a^	–	–

“–” indicates that there are no PCR-positive samples available for further determination of infectious MPXV using PMA-qPCR assay

^a^Means that the 6 samples were PCR-positive domestic wastewater from patients' living environments, and no infectious virus was detected above the PMA-qPCR detection limit

### Decay kinetics of monkeypox virus in wastewater

**Fig. 4:**
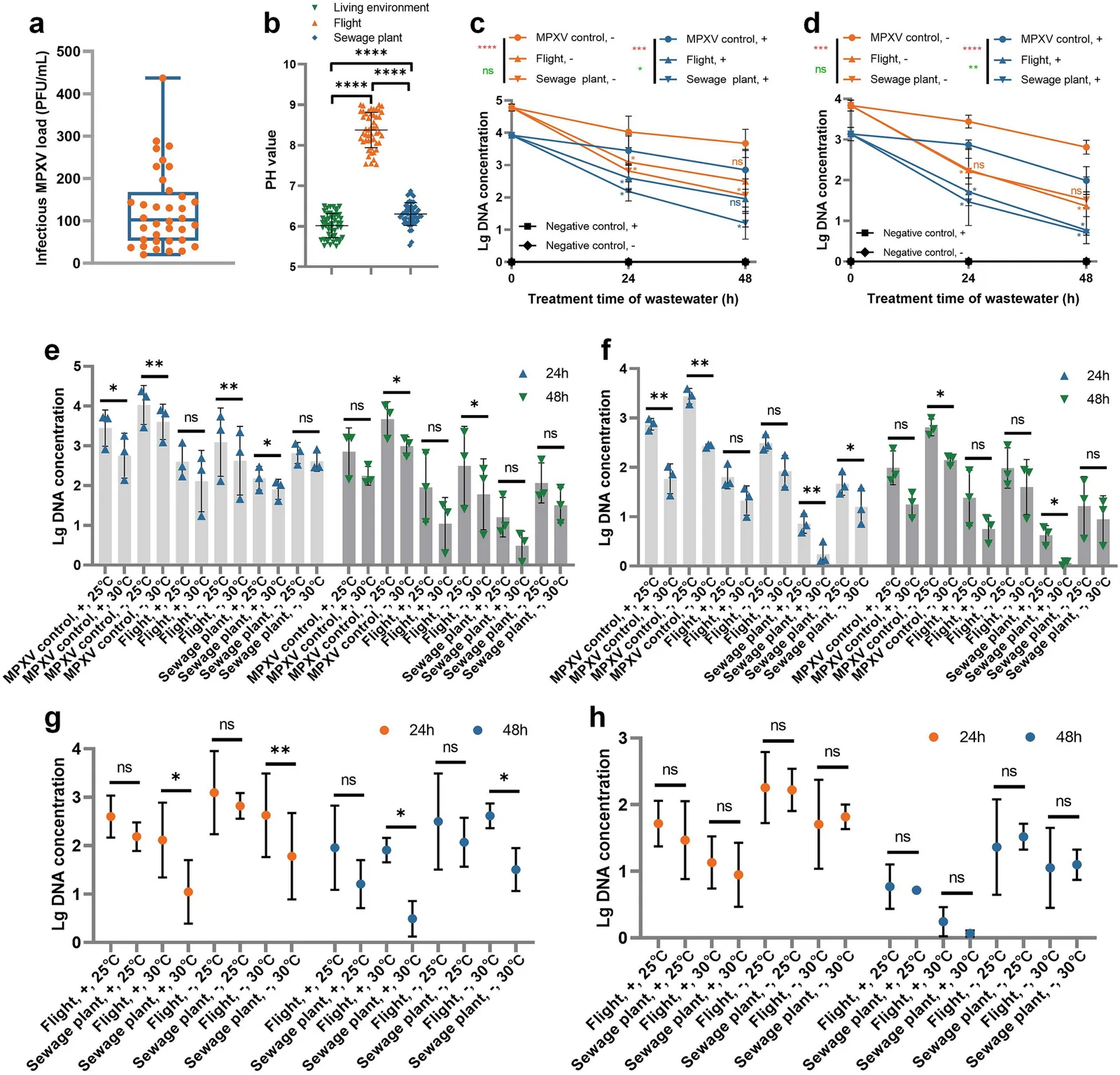
Decay kinetic characteristics of monkeypox virus entering different terminal domestic wastewater. **a** Infectious MPXV load of 36 surface samples of objects positive for PMA-qPCR assay. **b** pH distribution of different wastewater sources. Left box: green symbol, domestic wastewater from patients' living environments. Middle box: orange symbol, flight wastewater. Right box: blue symbol, sewage treatment plant wastewater. **c**, **d** The time decay kinetics of total MPXV DNA and infectious MPXV after adding MPXV with initial viral loads of 10^4^ and 10^3^ PFU/mL to wastewater. The negative control group (wastewater containing only culture medium) was used to verify the interference of wastewater impurities on PCR, and the results showed negative for all. **e**, **f** The effect of changes in environmental temperature on the decay of MPXV with initial viral loads of 10^4^ and 10^3^ PFU/mL added to wastewater. **g**, **h** The effect of different pH values of wastewater from different sources on the decay of MPXV with initial viral loads of 10^4^ and 10^3^ PFU/mL added to the wastewater

Further use of PMA-qPCR on the PCR-positive samples revealed that 25.71% (36/140) of the surface objects detected infectious MPXV, but the viral load was low with mean ± SD of 126.79 ± 93.57 PFU/mL, and the infectious MPXV load in domestic wastewater was below the detection limit and no positivity was observed (Fig. 4a and Table S6). To define practical monitoring windows for the Guangdong surveillance program, we conducted decay kinetics experiments to explore MPXV DNA and infectious MPXV loads after virus enters terminal domestic wastewater. We found that wastewater from different collection sources had significantly different pH ranges, with flight wastewater being alkaline with a mean of 8.376, while wastewater from sewage treatment plants and patient living environments was acidic with mean values of 6.301 and 6.018, respectively (Fig. 4b).

After entering the wastewater, both total MPXV DNA and infectious MPXV decayed more rapidly within 48 h compared to MPXV without added wastewater, showing a significant time decay effect. This decay effect was more significant in the continuous repeated monitoring of infectious MPXV, with the detected load at 24 and 48 h being more than one exponential level lower than MPXV without added wastewater. When the initial concentration was 10^3^–10^4^ PFU/mL, the infectious MPXV load rapidly decreased to 10–100 PFU/mL after 48 h of entering the wastewater, with a decay rate exceeding 99%. The attenuation of total MPXV DNA was relatively delayed, and a high load could still be detected after 48 h of entering the wastewater (Fig. 4c and d). Further verification indicated that the increase in environmental temperature and the acidity or alkalinity of wastewater were important factors driving the rapid decay of MPXV. Specifically, as the ambient temperature increased from 25 to 30 ℃, the detection load of MPXV DNA and infectious MPXV further significantly decreased (Fig. 4e and f), while the attenuation in acidic treatment plant wastewater was more significant than in alkaline flight wastewater (Fig. 4g and h). However, it is worth noting that there was no significant difference in the detected loads of MPXV DNA and infectious MPXV in the two different pH wastewater treatments at an ambient temperature of 25 ℃ or an initial viral load of 10^3^ PFU/mL. Only at an initial viral load of 10^4^ PFU/mL and an ambient temperature of 30 ℃, the infectious MPXV load in acidic wastewater was lower than that in alkaline flight wastewater, while there was no significant difference in total MPXV DNA (Fig. 4g and h).

## Discussion

During the ongoing global mpox outbreak, real-time monitoring of infectious virus across clinical and environmental settings has become essential for public health response. Standard qPCR, the WHO-recommended diagnostic gold standard, detects viral genetic material but cannot distinguish intact infectious virions from residual viral DNA derived from inactivated particles. This limitation complicates clinical management and environmental risk assessment, as positive PCR results may trigger unnecessary isolation when no infectious virus is present [31]. Our Guangdong surveillance data demonstrate that this distinction is clinically critical. Infectious MPXV loads were highest in skin lesions and anal swabs, exceeding throat swabs by more than 100-fold. These findings indicate that direct mucosal contact with lesions or secretions represents the predominant transmission route, whereas respiratory and indirect fomite-mediated routes pose minimal risk in practice. The low infectious viral load in throat swabs also supports this observation. Recent cohort studies and meta-analyses have consolidated transmission parameters from the 2022–2023 outbreak and confirmed that intimate physical contact remains the dominant route of spread [24, 28, 32, 33], and our results further support this conclusion. However, these observations are derived from a relatively small cohort of 15 patients with 23 clinical samples, and the uneven distribution of sample types across patients introduces variability. While the pattern is consistent with larger published cohorts, it should be interpreted as preliminary and requires confirmation in larger studies.

We established a PMA-qPCR assay that achieves concordance with the gold standard plaque assay for infectious virus quantification while requiring only 20 min of sample pretreatment. This rapid processing is valuable in outbreak settings where conventional cell culture methods are impractical due to lengthy incubation and BSL-3 requirements. The assay quantifies DNA from intact, membrane-enveloped particles. This principle has been established across bacteria [29], SARS-CoV-2 [30], and other enveloped viruses [34, 35], and operates by SDS facilitating PMA penetration of compromised envelopes, blocking amplification of non-infectious DNA while leaving DNA from infectious virions available for amplification. Membrane-destabilizing agents such as Triton X-100, Tween 20, and deoxycholate have been used in prior PMA-based assays, but SDS offers effective enhancement of dead viral envelope permeability with minimal impact on downstream DNA extraction and PCR efficiency [29, 30]. More importantly, the 2024–2025 resurgence of clade Ib in Africa [36] has renewed attention to the need for adaptable, deployable surveillance tools. Because our PMA-qPCR assay requires only primer replacement to address emerging strains, it aligns with the practical demands of responding to evolving outbreak scenarios.

Our environmental surveillance revealed patterns of viral shedding and persistence. Although MPXV DNA was detectable on 41.5% of patient-contact surfaces and in 11.3% of domestic wastewater, infectious virus was present at low levels on surfaces (mean 126.8 PFU/mL) and fell below detection limits in domestic wastewater. No MPXV DNA was detected in terminal wastewater from flights or sewage treatment plants, suggesting that dilution, transit time, and in-transit inactivation eliminate detectable signal before reaching downstream sampling points. These findings align with previous reports that environmental persistence depends on surface type, matrix composition, and ambient conditions [11, 17, 37]. The low infectious loads on household surfaces likely represent transient rather than sustained contamination, supporting routine disinfection of high-touch surfaces.

The decay kinetics experiments provide mechanistic insight into these observations. Infectious MPXV underwent more than 99% inactivation within 48 h in wastewater, with decay accelerated by acidic pH and elevated temperature [11]. The absence of detectable DNA at sewage treatment plants, despite sporadic local cases, suggests that dilution, biological degradation, and disinfection in municipal systems limit downstream detection. Nevertheless, high initial concentrations can still yield low levels of infectious virus at 48 h, particularly under cooler or less acidic conditions. This residual viability raises occupational health concerns, as sanitation workers exposed to untreated wastewater may face exposure risks, warranting continued biosafety precautions [11, 38].

Previous wastewater surveillance studies have relied on DNA detection alone, which cannot differentiate infectious virus from residual nucleic acid [19–23]. This ambiguity is problematic when high DNA loads trigger alerts that overestimate transmission risk. By integrating PMA-qPCR within the Guangdong CDC surveillance network, we demonstrate that infectious virus quantification provides operationally relevant data that DNA-based methods cannot offer. The assay provides a practical 48-h assessment window, informs isolation duration based on infectivity rather than DNA persistence, and guides targeted environmental monitoring during active outbreaks.

Furthermore, we acknowledge that the higher LOD of PMA-qPCR (10–100 PFU/mL) relative to standard qPCR (1–10 PFU/mL) represents an analytical limitation that must be considered when interpreting negative results in environmental samples, particularly in highly diluted wastewater. However, several independent observations in our study support the interpretation that infectious MPXV is genuinely scarce or absent in these matrices rather than merely below analytical detection. First, the assay successfully detected infectious virus on surface samples at comparable low titers (mean, 126.79 PFU/mL). Second, controlled decay experiments demonstrated that infectious MPXV decreases by more than 99% within 48 h in wastewater, providing a mechanistic basis for the dissociation between DNA detection and infectious virus detection. Third, terminal wastewater samples from flights and sewage treatment plants were entirely negative even by standard qPCR, indicating that viral DNA itself was undetectable at downstream sampling points. Consequently, while we cannot exclude the possibility that trace amounts of infectious virus below the LOD persist in some environmental samples, our negative findings are biologically plausible and consistent with the rapid inactivation kinetics observed under controlled conditions.

This study has limitations. The lack of detailed records on object types prevented analysis of MPXV carriage across surface categories. Flight and sewage treatment plant samples were collected from areas with reported cases but without confirmed patient linkage, potentially affecting detection sensitivity. In addition, real-world environmental matrices contain complex microbial communities and chemical constituents that may theoretically interfere with PMA penetration or PCR efficiency in ways not fully captured by our laboratory controls, particularly in continuous-flow wastewater systems. And these constituents contained in wastewater may also either protect virions or accelerate inactivation, indicating that our observed decay rates should be considered a baseline because actual environmental persistence may differ. Furthermore, the optimization of PMA and SDS concentrations was performed using NTV rather than MPXV, due to biosafety requirements. While NTV shares the same family and envelope architecture with MPXV, subtle differences in membrane permeability may exist between vaccinia-based vaccine strains and wild-type MPXV. PMA-qPCR has a higher detection limit than standard qPCR, meaning trace infectious MPXV in highly diluted wastewater may escape detection. Negative results should be interpreted as levels below the detection threshold rather than absolute absence.

## Conclusions

This study establishes PMA-qPCR as a practical tool for outbreak surveillance and provides a comprehensive characterization of infectious MPXV across the human-to-environment transmission spectrum in Guangdong, China. Infectious viral loads appeared highest in skin lesions and anal swabs, exceeding throat swabs by more than 100-fold. This pattern suggests that direct contact with mucosal lesions or secretions may represent the predominant transmission route, whereas respiratory and indirect fomite-mediated exposure poses minimal risk, though confirmation in larger studies is needed. In environmental matrices, infectious MPXV is detectable on patient-contact surfaces at low levels, but undergoes rapid decay in wastewater, with more than 99% inactivation within 48 h under typical conditions. The absence of infectious MPXV in PCR-positive domestic wastewater and terminal sewage underscores the critical distinction between nucleic acid detection and viable viral burden. By embedding this assay within the CDC surveillance network, we demonstrate that PMA-qPCR can simultaneously validate assay performance and generate actionable outbreak intelligence, providing evidence-based monitoring windows, informing isolation protocols, and guiding environmental surveillance strategies during active mpox epidemics.

## Supplementary Information

Supplementary material 1.

Supplementary material 2.

Supplementary material 3.

Supplementary material 4.

Supplementary material 5.

Supplementary material 6.

## Data Availability

No datasets were generated or analyzed during the current study.

## Abbreviations

MPXV: Monkeypox virus
PHEIC: Public Health Emergency of International Concern
WHO: World Health Organization
qPCR: Quantitative polymerase chain reaction
PMA-qPCR: Propidium monoazide-assisted quantitative polymerase chain reaction
SDS: Sodium dodecyl sulfate
CDC: Center for Disease Control and Prevention
NTV: Non-replicating Tiantan vaccinia virus
BSL-3: Biosafety level-3
CT: Cycle threshold
LOD: Limit of detection
