# Supplementary material for: From clinical fluids to environmental matrices: quantitative monitoring of infectious monkeypox virus based on PMA-qPCR during the Guangdong outbreak
Source: Trop Med Health. 2026 Jul 15;54:162. doi: 10.1186/s41182-026-01028-z (PMC13491699; doi:10.1186/s41182-026-01028-z)
Supplement: Supplementary file 1 [file 41182_2026_1028_MOESM1_ESM.docx]

**Supplementary Materials for**

From Clinical Fluids to Environmental Matrices: Quantitative Monitoring of Infectious Monkeypox Virus Based on PMA-qPCR During the Guangdong Outbreak

Jianhai Yu, Xin Zhang, Xiaoting Xie, Yutian Miao, Shen Huang, Li Zhu, Yan Zhan, Yifan Lin, Changyun Sun, Linqing Wang, Penghui Jia, Qinghua Wu, Weiwei Xiao, Bao Zhang, Chenguang Shen, Baisheng Li and Wei Zhao

Correspondence to: [zhaowei@smu.edu.cn](mailto:zhaowei@smu.edu.cn) (W.Z.); [libsn@126.com](mailto:libsn@126.com) (B.L.) and [a124965468@smu.edu.cn](mailto:a124965468@smu.edu.cn) (C.S.).

**This PDF file includes:**

Figure S1. Correspondence between two naming methods of MPXV encoded proteins and their functional annotation.

Table S1. Primers and probes for qPCR targeting OPG95, OPG104, and OPG143 genes encoding M1R, L5L, and A17L for detecting MPXV.

**Other Supplementary Materials for this manuscript include the following:**

Table S2. Background information of virus cell cultures and mpox patient samples used in this study.

Table S3. Summary of gene localization and functional annotation information of complete coding proteins of MPXV.

Table S4. Public monkeypox virus complete genome sequences used for alignment analysis.

Table S5. List of environmental sample information for inbound flights at Guangzhou Baiyun Airport.

Table S6. Collection and testing results of object surfaces and wastewater samples in the living environment of 59 mpox patients.

**Figure S1.** Correspondence between two naming methods of MPXV encoded proteins and their functional annotation. Five functional protein groups were identified according to the types of biological functions performed, namely Group I: influencing viral infection and pathogenesis through host regulation; Group II: participating in the formation, assembly, budding and release of viral particles; Group III: surface proteins of viral particles mediating host infection; Group IV: regulating the replication and translation of the viral genome; Group V: Proteins marked as unknown by functional annotation.

**Table S1.** Primers and probes for qPCR targeting OPG95, OPG104, and OPG143 genes encoding M1R, L5L, and A17L for detecting MPXV.

| **Gene** | **Nucleotide sequence** | **Sequence length (bp)** |
| --- | --- | --- |
| M1R |  |  |
| Forward primer | CCACTACTCAAATAGCAC | 18 |
| Reverse primer | GCTTGGCATAGTACATAA | 18 |
| Probe | ACGCTGCCAATATTATAACACCGAT | 25 |
| A17L |  |  |
| Forward primer | CCCGGATGCTATATAATCA | 19 |
| Reverse primer | TGGAGAACCATGTAGTTC | 18 |
| Probe | TCAGGAGTTACCTCGTTCTGATAAT | 25 |
| L5L |  |  |
| Forward primer | CATCGCTTCGTTTACATG | 18 |
| Reverse primer | GACGATATACGATGTAAATGTA | 22 |
| Probe | AGGGTAATCTTGTATCTATTCCTATCCGT | 29 |
